# Differentiated Anti-Predation Responses in a Superorganism

**DOI:** 10.1371/journal.pone.0141012

**Published:** 2015-11-11

**Authors:** Thomas A. O’Shea-Wheller, Ana B. Sendova-Franks, Nigel R. Franks

**Affiliations:** 1 School of Biological Sciences, University of Bristol, Life Sciences Building, 24 Tyndall Avenue, Bristol, England; 2 Department of Engineering Design and Mathematics, UWE Bristol, Frenchay Campus, Coldharbour Lane, Bristol, England; Universidade de São Paulo, Faculdade de Filosofia Ciências e Letras de Ribeirão Preto, BRAZIL

## Abstract

Insect societies are complex systems, displaying emergent properties much greater than the sum of their individual parts. As such, the concept of these societies as single ‘superorganisms’ is widely applied to describe their organisation and biology. Here, we test the applicability of this concept to the response of social insect colonies to predation during a vulnerable period of their life history. We used the model system of house-hunting behaviour in the ant *Temnothorax albipennis*. We show that removing individuals from directly within the nest causes an evacuation response, while removing ants at the periphery of scouting activity causes the colony to withdraw back into the nest. This suggests that colonies react differentially, but in a coordinated fashion, to these differing types of predation. Our findings lend support to the superorganism concept, as the whole society reacts much like a single organism would in response to attacks on different parts of its body. The implication of this is that a collective reaction to the location of worker loss within insect colonies is key to avoiding further harm, much in the same way that the nervous systems of individuals facilitate the avoidance of localised damage.

## Introduction

Colonies of social insects can achieve extraordinary levels of collective organisation, and as such, they are often referred to as single ‘superorganisms’ [1]. This may be manifest in evolutionary terms; certain insect societies can be single units of selection [2], or in physiological terms; with some social insect colonies displaying caste polymorphism which mirrors cell specialisation in individuals [3–5]. Furthermore, collective behaviour may confer spatially specific advantages in response to threats such as predation at the group level. In single organisms, antagonistic stimulation has markedly contrasting effects depending on its location. This is exemplified by the *Xenopus* tadpole, that, when touched on the tail, will flex it in the opposite direction to the stimulus, but when touched on the head, will swim away in a random direction [6]. Similarly, the scallop *Euvola ziczac* will ‘jump’ to avoid starfish that come into contact with its outer shell mantle, but perform longer escape swims when a starfish touches its dorsal ears [7]. Simple but specific behaviours such as these are effective in mitigating risks to the individual, and as such, it may be advantageous for colonies of social insects to react differentially to predation in an analogous fashion.

The use of alarm pheromones by ants is well documented. Some species employ both ‘aggressive’ alarms, to draw nest mates towards potential intruders, and ‘panic’ alarms; to facilitate evasion or evacuation when faced with an insurmountable foe [8]. Another strategy, seen in the weaver ant *Oecophylla longinoda*, involves both chemical and physical cues; alarm pheromones will excite major workers to attack a predator, while causing minor workers to remain within the nest, and only if the nest itself is then disturbed will the minors respond [9]. While such examples demonstrate the complexity of colony-level reactions to predation, they make use of chemical rather than tactile signals, and often elicit responses dependent on the proximity to a pheromone source, rather than the location of the pheromone within the colony [1].Thus, to understand better how social insects react to differing types of predation, it may be advantageous to assess this in a species where predation can be simulated at discrete loci within the colony.

The ant *Temnothorax albipennis* provides a suitable model with which to do this, as specific individuals can be removed from different parts of colonies, enabling the experimental assessment of varying forms of perturbation. Furthermore, in this genus, it has been well-established that the spatial organisation of workers within a colony is strongly linked to their roles, and thus it is possible to target and manipulate workers with known task propensities [10, 11]. This species will also migrate to nests of better quality even if their current nest is intact, in so-called move-to-improve emigrations [12]. The emigration process itself provides a unique tool with which to assess colony-level responses to predation, as differing behaviours may be manifest in the rate of emigration to new nests that would otherwise be missed under normal circumstances.

There is evidence to suggest that ants of this species are able to monitor mortality risk outside the nest. In the experiments of Richardson et al. [13], scouting workers leaving the nest were removed sequentially and the interval measured until the next scout appeared. In the control, individually marked scouts were allowed to return to the nest and the time taken for the next ‘new ant’ (i.e. one never seen outside of the nest before) to emerge was measured. In such controls, the time taken for each new ant to exit the nest increased over the observation period. This is to be expected given the well-known division of labour in such ants; some workers will mostly stay within the nest while others will habitually go outside, and given that the transition from working inside to working outside is a slow and regulated process. However, when scouts were removed upon emergence from the nest, the exit rate of new scouts was even slower, indicating that those within the nest were actively avoiding going outside due their nest mates’ failure to return. This process is likely made possible through the spatial structure of the workforce in these colonies, as workers that are prone to leave the nest have spatial fidelity zones near the nest entrance, and thus can monitor outgoing and incoming traffic [10, 11]. In conjunction with this, a recent study using a closely related species; *Temnothorax rugatulus*, has found that during migrations, ants respond differently to the alarm pheromone 2, 5-dimethylpyrazine (DMP) dependent upon its perceived context [14]. Taking this into account, it seems likely that *T*. *albipennis* may also make use of context in shaping colony responses to threat.

Previous experiments have also shown that when the nest is destroyed, causing forced emigration, colonies can still choose the better and more distant of two nest sites presented to them [15]. Here we employ the same design but do not destroy the current nest, instead we subject the ants to various predation scenarios. As such, we are able to assess the effects of predation both upon the rate of migration and upon final nest choice. In our experiments, we remove individuals from the periphery of colony scouting activity, and from within the nest itself, simulating predation targeted at very different parts of the colony. Additionally, we compare these scenarios to that of an emergency emigration, in which the original nest is destroyed, using data from [15] and a control treatment, in which no ants are removed. Using these techniques, we then assess the varying responses of the colony during migration to a new nest.

## Materials and Methods

### a) Colonies

We collected 30 colonies of *Temnothorax albipennis* from the isle of Portland, Dorset (50.547889°, -2.448251°) on 20th January 2014, each containing a queen and from 12 to 268 workers. No permission for collection was required, as colonies were taken from a heavily disturbed quarry area open to the public. However, although *T*. *albipennis* is not a protected species, all care was taken to minimise disturbance of the population by adherence to a rota of varied collection (based on date and location). Colonies were maintained under standard laboratory conditions, and all nests were housed in plastic Petri dishes with Fluon coated sides. Ants were fed with *Drosophila melanogaster* weekly, and allowed to forage for water and honey solution *ad libitum* [12]. This method of feeding was chosen in preference to the Batkar and Whitcomb diet, which is suggested to be unsuitable for species that consume both invertebrate prey and honeydew [16].

### b) Nest groups

Prior to the initiation of experimental trials, we grouped the 30 colonies into sets of three based on size to ensure that each group had an even size distribution, then randomly assigned each colony to one of three experimental manipulation groups: the control (median colony size: 132), the nest predation (median colony size: 123) and the peripheral predation group (median colony size: 96). All colonies were forced to emigrate into initial ‘poor’ nests, ten of which (nest predation treatment) had acetate lids composed of two layers; one with holes for ant removal and a second solid layer to act as a cover. While the covers of these nests were constructed differently from those of the other ‘poor’ quality nests, the two layers ensured that they had no increased air flow, and thus would not have been perceived by the ants as differing in quality from the other ‘poor’ nests. During a seven day period all colonies were left to acclimatise, none of the colonies housed in ‘poor’ nests made any attempt to emigrate. Eleven colonies were used in the nest destruction experiment.

**Fig 1:**
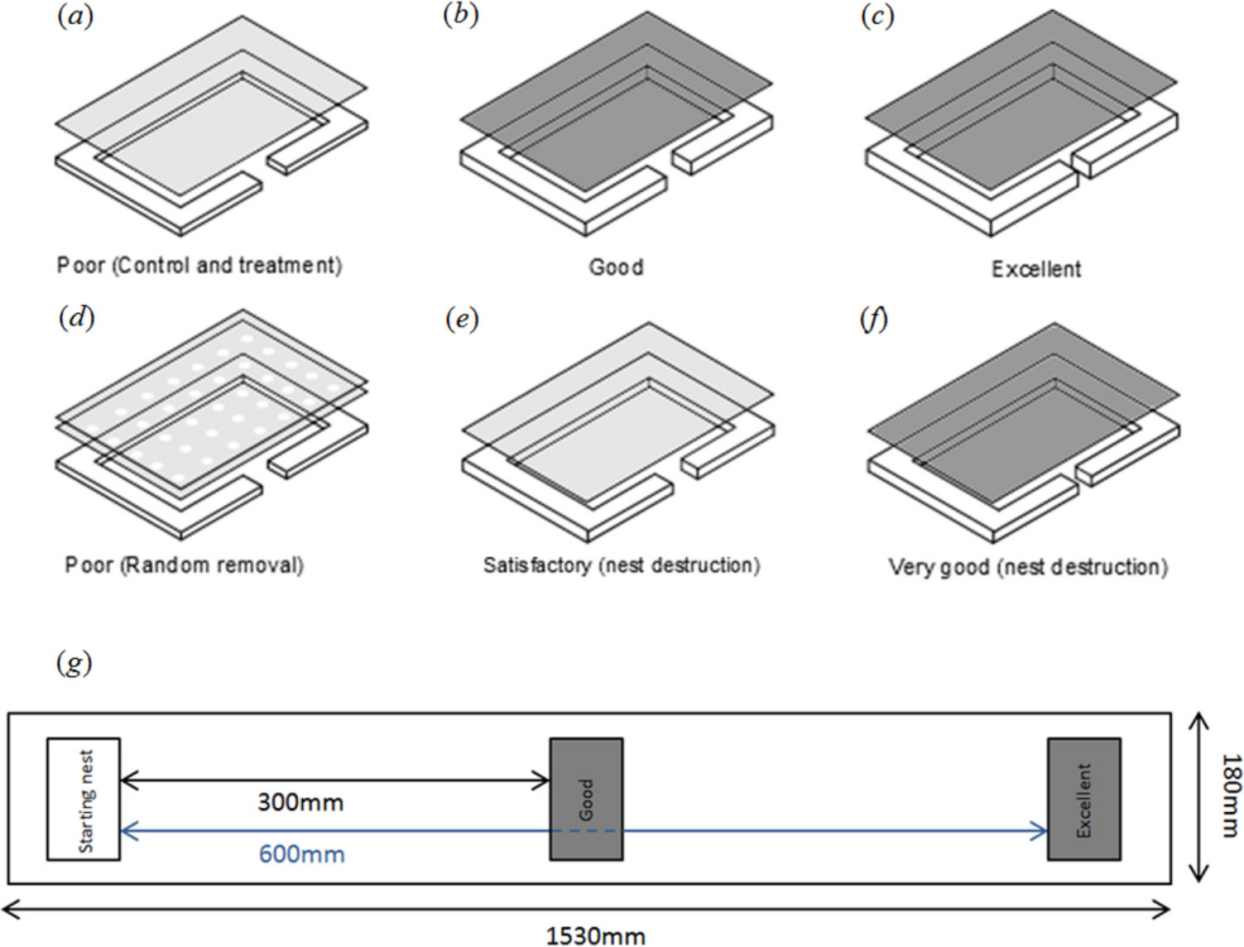
The range of nest types used in the experiment: (a) clear cover, 1mm wall height, 4mm entrance width; (b) red filter cover, 2mm wall height, 4mm entrance width; (c) red filter cover, 2mm wall height, 1mm entrance width; (d) clear cover, 1mm wall height, 4mm entrance width, acetate film with holes for ant removal and second acetate layer to act as a cover; (e) clear cover, 1.8mm wall height, 4mm entrance width; (f) red filter cover, 1.8mm wall height, 2mm entrance width. (g) Experimental arena, starting nests were of poor quality and were not destroyed, allowing voluntary emigrations to occur. A similar arena was used for the nest destruction treatment, measuring 800x165mm [15].

Overall we used five different nest qualities, in order of perceived quality these were; ‘poor’, ‘satisfactory’, ‘good’, ‘very good’ and ‘excellent’ [17]. In our present experiment we used ‘poor’, ‘good’ and ‘excellent’ nests, while in the earlier experiment ‘satisfactory’ and ‘very good’ nest qualities were used (Fig 1; [15]). Nests had an internal cavity area of 60×35mm in the control, nest predation and peripheral predation groups and 50×33 mm in the nest destruction group. Red filters were used to reduce light levels within the good, very good and excellent nests, creating an environment perceived by the ants as being darker and thus preferable [17, 18].

### c) Experimental protocol

Thirty emigrations were conducted in total for the current experiments, each using a different colony, between 2.00pm and 5.00pm, over a ten day period from 27^th^ January to the 5^th^ February 2014. All three manipulation groups were used each day, and experimental trials were performed within 1530×180mm rectangular plastic arenas with Fluon coated sides. To investigate rigorously the responses of colonies, we used a nest choice design in which the excellent new nest site was further away than the good nest site (Fig 1). This design increased the difficulty of making a choice between new nest sites, and was also used in the earlier experiment [15].

Before initiation of trials, using a fine-tipped brush we removed all scouting ants leaving the nests of the peripheral predation colonies during a 30-min period, simulating predator induced mortality. Immediately after this, again using a brush, we removed the same proportion of ants randomly from within the corresponding nest predation colonies. The removal process required the temporary lifting of the second solid portion of the nest lid in order to access the holed acetate; however nest integrity was maintained, as all four walls and cover remained intact and the colonies were not exposed. Removals accounted for an average worker reduction of 16% in peripheral predation and nest predation colonies. We ensured that the removal period for nest predation colonies never exceeded 4 min, in order to keep nest disturbance to a minimum, and after removal, the second solid lid layer was replaced in order to avoid any perceived changes in nest quality during the trial. Control colonies underwent no manipulation; however, as was the case in all treatments, any scouts outside the nests were placed on to the top of nests in order to avoid an initial scouting advantage. Timers for the separate manipulation groups were started, and in each arena, we placed a good and an excellent quality nest in the positions detailed in (Fig 1). Both good and excellent nests were included as previous work has shown that, under forced emigrations, colonies can successfully choose the better of two options at this distance [15]. We then recorded the number of ants in each nest every 10 min for the first 120 min of the emigration, and the first nest to be discovered by each colony. Emigrations were allowed to continue until their completion or up to a cut-off point of 24 h. After completion of trials, detailed photographs were taken of all nests and final nest choices were recorded.

The nest destruction trials were conducted in a previous study, in arenas of similar dimensions and with new nests at the same distances as in the present study; however the original nests were destroyed by removal of the glass lids, exposing the colonies and forcing the ants to migrate. During these earlier trials, worker build-up was measured in a Satisfactory nest (at 300 mm) and Good nest (at 600 mm) over time, using eleven colonies. For full experimental details see [15].

### d) Data analysis

**Fig 2:**
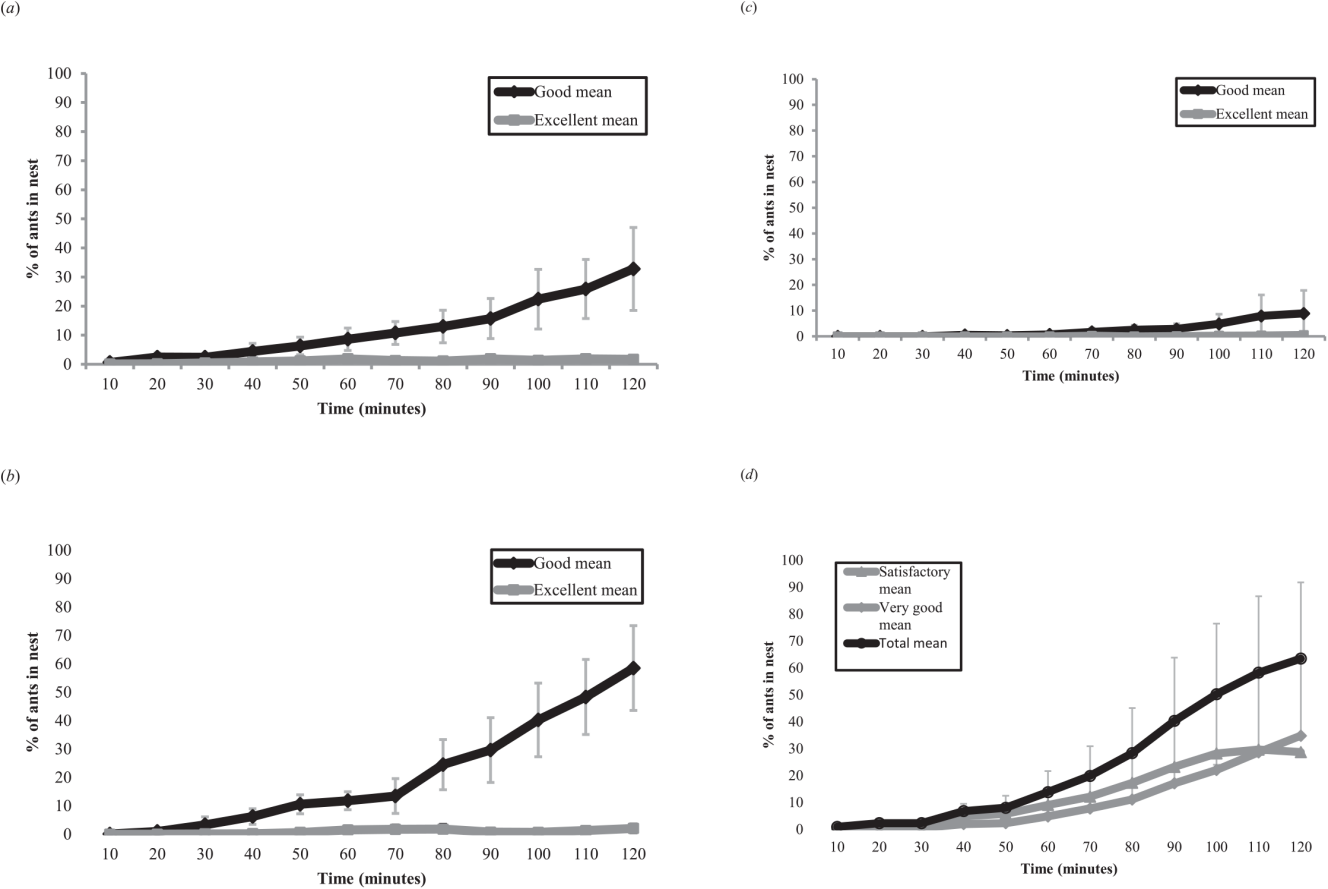
Mean percentage of total colony workers in nests of each quality over time for (a) control; (b) nest predation, (c) peripheral predation groups, and (d) when the original nest was destroyed (from previously published data). The mean represents n = 10 colonies for each time point in (a)–(c) and n = 11 colonies in (d). Error bars indicate 95% confidence intervals.

We used a General Linear Model to assess the effects of manipulation on mean numbers of ants in new nests over time, as a percentage of total colony size. The interaction between manipulation group and time was tested, with manipulation group as the fixed factor and time as the covariate. We conducted a Shapiro-Wilk test to confirm that the residuals were not significantly different from normal (Statistic = 0.986, df = 48, P = 0.839). We used a One-way ANOVA to analyse differences in the final percentages for each treatment group and a Turkey’s HSD test, with sample sizes of 12, to assess the pairwise comparisons. We used a 3×3 Fisher’s exact test to assess differences in final nest choice between manipulation groups, this was used as some categories contained no values, and thus a Pearson’s chi-squared test was not appropriate. The eleven colonies in the nest destruction group, all of which made the best choice, were not included in this analysis. All statistical analyses were performed in SPSS (Release version 21.0.0.0 IBM Corporation and other(s) 1989, 2012). In order to compare migration dynamics to those of an emergency emigration, data from an earlier experiment [15], using an analogous method, was utilised to generate (Fig 2D).

## Results

**Fig 3:**
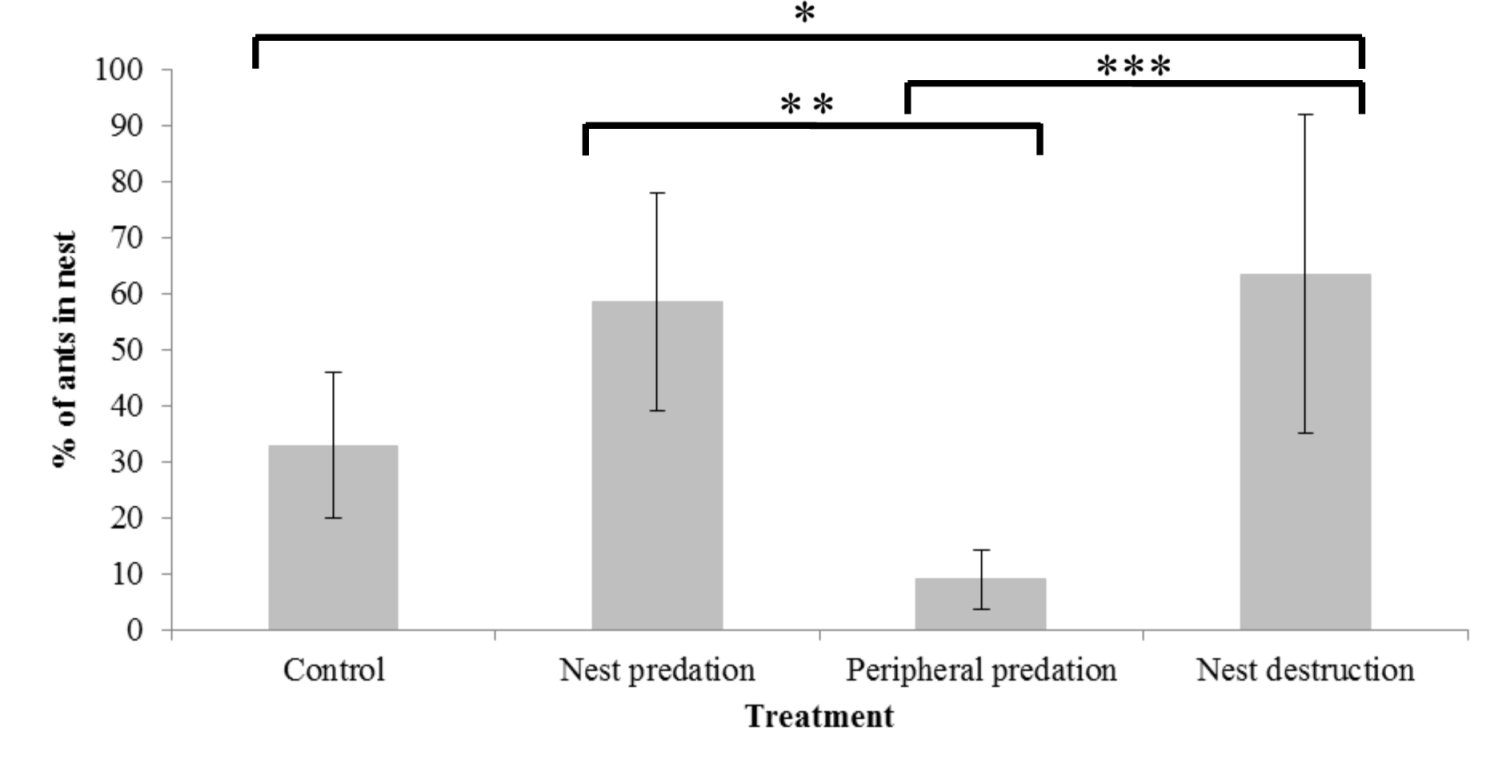
Mean percentage of total colony workers in nests at final time points (at 120 min). The mean represents ants in the good nests (n = 10 colonies) for the control, nest predation and peripheral predation groups and ants in both nests (n = 11 colonies) for the nest destruction group (from previously published data). Significant pair-wise comparisons between treatment group means are marked with asterisks (*-P<0.05, **-P<0.01, ***-P<0.001) other comparisons are not significant. Error bars indicate 95% confidence intervals.

The migration rate into good nests was reduced in the peripheral predation group, and increased in the nest predation group in comparison to the control group. In the nest destruction group, migration into both poor and good nests occurred and total migration rate was higher than in any other treatment (Fig 2). In all three ‘move-to-improve’ migrations, a negligible percentage of ants visited the excellent nests, thus we analysed only the percentage of ants moving to the good nests in these groups. In contrast to this, as ants visited both nests in the ‘emergency’ nest destruction migration, we also considered total migration rate to both new nests in this group. The two-way interaction between manipulation group and time from the start of the experiment was significant (GLM: F_3,40_ = 38.303, P = <0.001). This was due to differences in the accumulation rate of ants in good nests between manipulation groups; with more rapid accumulation in the nest predation and nest destruction groups, and slower accumulation within the peripheral predation group in comparison to the control (Fig 2). Specifically, posthoc pairwise comparisons showed significant differences in the rate of ant accumulation over time in good nests between the control and nest predation (t = 4.416, P<0.001), control and peripheral predation (t = -3.510, P = <0.001) and peripheral predation and nest predation groups (t = 7.926, P = <0.001). There was no significant difference between accumulation of ants in the good nest in the nest predation group, and the total accumulation of ants in both nests within the nest destruction group (t = 1.716, P = 0.094; Fig 2). The mean final percentage of ants in nests differed significantly between treatment groups (one-way ANOVA: F_3,40_ = 9.709, P = <0.001) (Fig 3). Posthoc pairwise comparisons showed significant differences between nest predation and peripheral predation (Mean difference = 49.547, Std. Error = 11.587, P = 0.001), peripheral predation and nest destruction (Mean difference = -54.524, Std. Error = 11.320, P = <0.001) and control and nest destruction groups (Mean difference = -30.670, Std. Error = 11.320, P = 0.048) (Fig 3).

Although in the move-to-improve migrations the effect of manipulation on final nest choice was not statistically significant, it is worth highlighting that all ten colonies in the nest predation group made a moderate choice while this was not the case in the peripheral predation and control groups (Fisher’s exact statistic = 7.044, P = 0.067, Table 1). By contrast, every one of the eleven nest destruction colonies made the best final nest choice (Table 1).

**Table 1:** Cross tabulation for effect of manipulation on final nest choice.

Manipulation group	Final nest choice			Total
	Impaired	Moderate	Best choice	
Control	1	6	3	10
Nest predation	0	10	0	10
Peripheral predation	3	6	1	10
Nest destruction	0	0	11	11
Total	4	22	15	41

Best choice: chose the better new nest, or split between the two new nests; moderate: chose the worse new nest; impaired: remained in starting nest or split between starting and worse new nests (nest destruction from previously published data).

## Discussion

Our results show a concerted anti-predation response consistent with the superorganism concept. Simulated predation of peripheral scouting workers markedly inhibited the progression of emigrations over time. This phenomenon was characterised by both later discovery of, and a slower build-up of scouting ants within the good nests in the peripheral predation group compared to the control, nest predation and nest destruction groups (Fig 2). In contrast, simulated predation of individuals from within the nest increased emigration speed. There was a significantly faster build-up of workers over time in the good nests within the nest-predation group, compared to the control and peripheral predation groups (Fig 2). This increased emigration rate was lower, but not statistically different to that triggered by an emergency emigration; however, the dynamics after worker removal from within the nest differed substantially from those when the nest was destroyed. Specifically, when the nest was destroyed, the number of workers built up at a similar rate in both new nests, suggesting that destruction of the nest led to a loss of colony cohesion (Fig 2D). By contrast, only two or three ants visited the excellent nest at any one time in the nest predation group, implying that the colony as a whole maintained its unity of choice (Fig 2B). Moreover, it seems unlikely that this difference in dynamics was due simply to scouting ants being ‘intercepted’ by the good quality nest in the nest predation treatment and then failing to upgrade to the excellent one. This is because the difference in quality and distance were the same for the control and peripheral predation groups, yet several colonies still migrated to the excellent nests. However, in the nest predation group, all ten experimental colonies moved to the good nest and were still there after 24 h (Table 1). Taken together, our results suggest that colonies will retract into their current nest in response to scout loss at the periphery, but vacate rapidly in response to predation of workers from within the nest, while maintaining a level of cohesion absent from emergency emigrations [19].

Furthermore, although we found that the effect of scout removal on final emigration choice up to 24 hours after the start of the experiment was not statistically significant; our results suggest that such an effect cannot be ruled out. Previous studies have shown that colonies will migrate to farther-away excellent nests under forced emigration conditions [15], but here we show that this is not the case during migrations under the influence of worker loss. These findings are consistent with a robust form of predator avoidance behaviour, implemented at the colony level, and there are several potential mechanisms by which this may occur. The loss of specialist scouts may play a role in retarding colony exploration levels; however studies have shown that *T*.*albipennis* colonies have highly flexible task allocation structures, so would potentially be able to replace lost scouts [20]. Moreover, as has been shown earlier [13], that worker exit rate under such predation conditions is significantly slower than would be expected in response to depletion of scout specialists, or as a consequence of the spatial organisation of tasks [10, 11]. In view of this, it seems more plausible that down-regulation is involved in reducing worker exits when nest mates do not return from scouting [13]. This process acts much in the same way as the withdrawal reflex in response to harmful stimuli in single organisms [21], and effectively avoids unnecessary mortality when predators are present outside the nest. In concert with this, the ‘evacuation’ response of colonies to predation targeted within the nest ensures that the vulnerable brood and queen are removed from harm. However, during the process, the colony retains its cohesion [19] and migrates directly into a single nest. In contrast, when the original nest is destroyed; colonies begin to migrate into both available new nests. As such it appears that two different threats, destruction of the nest, and loss of workers from within the nest, elicit comparable rates of evacuation but that the dynamics of worker accumulation in the new nests are different as the colony searches for a new home.

The underlying mechanism behind such nest-evacuation behaviour is likely multi-faceted. There is a possibility that the queen plays a role in regulating colony behaviour, as seen in some primitively eusocial insects [22]. However, in Temnothorax albipennis, the queen is always passively transported during migrations, and though this event usually takes place half way through any given emigration, the presence of a queen, in itself, is not essential in order for migrations to occur [23]. Pheromones may well be involved, as seen in the closely-related species *T*. *rugatulus*. In this species, when workers release the alarm pheromone 2, 5-dimethylpyrazine (DMP) in a dangerous environment distant from the colony, nest mates will avoid the area, while when the same pheromone is released within their home nest, workers are instead attracted in order to mount a defence [14]. In this scenario, context plays a central role in colony responses to threat signals. However, our results suggest that if similar signals are used in *T*.*albipennis*, they elicit a very different response when released within the home nest, leading to aversion rather than attraction. Although it might be argued that removal of ants from within the nest is a highly artificial scenario, the perceived threat it poses is likely tantamount to attacks by slave-raiding ants or invertebrate predators, and thus it is not unreasonable that ants in this genus may have evolved specific solutions [24]. Furthermore, such a response is consistent with other species that have small colony sizes, for which escape is often the preferable option [25], especially in cases where many potential new nest sites are present [26]. Additionally, as scout mortality may occur over a large area, the use of alarm pheromones to stop workers leaving the nest could prove unfeasible. In view of this, it is tempting to speculate that *T*. *albipennis* makes use of both the down-regulation of record dynamics to ameliorate scout losses [13], and alarm pheromones in order to facilitate rapid and cohesive nest evacuation when faced with a threat in their current home. Jointly, these two behaviours would make for a fluid and reactive solution to the issue of predation, whereby feedback throughout the colony elicits action by all of its members, regardless of their individual exposure to the threat.

Analogous systems that make use of modularised threat responses can be observed in numerous other unrelated taxa [6, 7]. In some species, responses may be modularised in relation to threat identity, rather than location, such as in the differing responses of the Cicada killer wasp *Sphecius Speciosus* to territorial invaders, and the reactions of Polistine wasps to predators [27, 28]. Additionally, parallels may be seen between the functioning of *Temnothorax albipennis* colonies, and that of individual vertebrates. One such example is the organisation of neurones responsible for the c-start escape reflex in teleost fish [29]. This process is thought to be controlled by two neuronal groups, sometimes referred to as the A1 and A2 groups. When a predator approaches from the posterior, the A1 neurones are maximally activated and the fish performs a turn away from the predator, while when a predator approaches from the anterior, both sets of neurones are activated, causing the fish to escape in the opposite direction [29]. The reactions of two different groups of workers in our experiments; those scouting and those within the nest, act in a similar way to these two groups of neurons; they illicit a response specifically appropriate to harm directed at their part of the colony.

Our findings further elucidate the mechanisms that facilitate anti-predator behaviour within social insects, and concur with previous studies that suggest *T*.*albipennis* colonies are robust to worker loss [30]. Moreover, we have found that as in many other aspects, superorganisms may benefit from reacting as a single entity to the threat of predation. This highlights the propensity for ant colonies to employ a multi-organismal ‘nervous system’ to deal with challenges, and provides rich potential for future studies.

## Supporting Information

S1 Data Raw data from the experiment. (XLSX)
